# Efficient Regularized Regression with *L*_0_ Penalty for Variable Selection and Network Construction

**DOI:** 10.1155/2016/3456153

**Published:** 2016-10-24

**Authors:** Zhenqiu Liu, Gang Li

**Affiliations:** ^1^Samuel Oschin Comprehensive Cancer Institute, Cedars-Sinai Medical Center, Los Angeles, CA 90048, USA; ^2^Department of Biostatistics, School of Public Health, University of California at Los Angeles, Los Angeles, CA 90095-1772, USA

## Abstract

Variable selections for regression with high-dimensional big data have found many applications in bioinformatics and computational biology. One appealing approach is the *L*_0_ regularized regression which penalizes the number of nonzero features in the model directly. However, it is well known that *L*_0_ optimization is NP-hard and computationally challenging. In this paper, we propose efficient EM (*L*_0_EM) and dual *L*_0_EM (D*L*_0_EM) algorithms that directly approximate the *L*_0_ optimization problem. While *L*_0_EM is efficient with large sample size, D*L*_0_EM is efficient with high-dimensional (*n* ≪ *m*) data. They also provide a natural solution to all *L*_*p*_ *p* ∈ [0,2] problems, including lasso with *p* = 1 and elastic net with *p* ∈ [1,2]. The regularized parameter *λ* can be determined through cross validation or AIC and BIC. We demonstrate our methods through simulation and high-dimensional genomic data. The results indicate that *L*_0_ has better performance than lasso, SCAD, and MC+, and *L*_0_ with AIC or BIC has similar performance as computationally intensive cross validation. The proposed algorithms are efficient in identifying the nonzero variables with less bias and constructing biologically important networks with high-dimensional big data.

## 1. Introduction

Variable selection with regularized regression has been one of the hot topics in machine learning and statistics. Regularized regressions identify outcome associated features and estimate nonzero parameters simultaneously and are particularly useful for high-dimensional big data with small sample sizes. Big data are datasets with either huge sample size or very high dimensions or both. In many real applications, such as bioinformatics, image and signal processing, and engineering, a large number of features are measured, but only a small number of features are associated with the dependent variables. Including irrelevant variables in the model will lead to overfitting and deteriorate the prediction performance. Therefore, different regularized regression methods have been proposed for variable selection and model construction. *L*_0_ regularized regressions, which directly penalize the number of nonzero parameters, are the most essential sparsity measure. Several popular information criteria, including Akaike information criterion (AIC) [1], Bayesian information criterion (BIC) [2], and risk inflation criteria (RIC) [3], are based on *L*_0_ penalty and have been used extensively for variable selections. However, solving a general *L*_0_ regularized optimization is NP-hard and computationally challenging. Exhaustive search with AIC or BIC over all possible combinations of features is computationally infeasible with high-dimensional big data.

Different alternatives have been proposed for the regularized regression problem. One common approach is to replace *L*_0_ by *L*_1_. *L*_1_ is known as the best convex relaxation of *L*_0_. *L*_1_ regularized regression [4] is convex and can be solved by an efficient gradient decent algorithm. It has found many applications in statistical genetics, bioinformatics, and medicine [5, 6]. Minimizing *L*_1_ is equivalent to minimizing *L*_0_ under certain conditions. However, the estimates of *L*_1_ regularized regression are asymptotically biased, and lasso may not always choose the true model consistently [7]. Experimental results by Mancera and Portilla [8] also posed additional doubt about the equivalence of minimizing *L*_1_ and *L*_0_. Moreover, there were theoretical results [9] showing that while *L*_1_ regularized regression never outperforms *L*_0_ by a constant, in some cases *L*_1_ regularized regression performs infinitely worse than *L*_0_. Lin et al. [9] also showed that the optimal *L*_1_ solutions are often inferior to *L*_0_ solutions found using greedy classic stepwise regression, although solutions with *L*_1_ penalty can be found effectively. More recent approaches aimed to reduce bias and overcome discontinuity include SCAD [10], *L*_*p*_ *p* ∈ (0,1] regularization [11, 12], and MC+ [13]. However, multiple free parameters (*λ* and *p*) must be tuned in those approaches, which is computationally intensive. They are not suitable for big data mining. Even though there are some effects for solving *L*_0_ regularized optimization problems [14, 15], *L*_0_ was either approximated by a different continuous smooth function or transformed into a much larger ranking optimization problem. To the best of our knowledge, currently, there is no efficient method directly approximating *L*_0_ for big data problem.

In this paper, we propose efficient EM algorithms that directly approximate *L*_0_ regularized regression problem. Our proposed approaches effectively deal with *L*_0_ optimization by solving a sequence of convex *L*_2_ optimizations and are efficient for high-dimensional data. They also provide a natural solution to all *L*_*p*_ *p* ∈ [0,2] problems, including lasso with *p* = 1, elastic net with *p* ∈ [1,2] [16], and the combination of *L*_1_ and *L*_0_ with *p* ∈ (0,1] [17]. Similar to lasso, the regular parameter *λ* can be determined by the generalized information criterion [18]; optimal *λ* with *L*_0_ regularized regression can also be predetermined with different model selection criteria such as AIC, BIC, and RIC. *L*_0_ local graphical model with either AIC or BIC is faster than *L*_1_ with cross validation. We demonstrate our methods through simulation and high-dimensional genomic data. The proposed methods identify the nonzero variables with less bias and outperform lasso, SCAD, and MC+ by a large margin. They can also choose the important genes and construct biological networks effectively.

## 2. Methods

Given an *n* × 1 dependent variable **y** and an *n* × *m* feature matrix *X*, a linear model is defined as

(1) $$\begin{array}{c} y=X\theta +\varepsilon , \end{array}$$

where *n* is the number of samples and *m* is the number of variables and *n* ≪ *m*, *θ* = [*θ*_1_,…, *θ*_*m*_]^*t*^ are *m* parameters to be estimated, and *ε* ~ *N*(0, *σ* ^2^ *I*_*n*_) are the random errors with mean 0 and variance *σ* ^2^. Assume that only a small subset of {**x**_*j*_}_*j*=1_^*m*^ has nonzero *θ*_*j*_s. Let *R*⊆{1,…, *m*} be the subset index of relevant variables with *θ*_*j*_ ≠ 0, and let *O*⊆{1,…, *m*} be the index of irrelevant features with 0 coefficients; we have *R* ∪ *O* = {1,2,…, *m*}, *X*_*R*_ ∪ *X*_*O*_ = *X*, and *θ*_*R*_ ∪ *θ*_*O*_ = *θ*, where *θ*_*O*_ = 0. The error function for *L*_0_ regularized regression is

(2) E12y−Xθ2+λ2θ0=12∑i=1nyi−∑j=1mθjxij2+λ2∑j=1mIθj≠0,

where ‖*θ*‖_0_ = ∑_*j*=1_^*m*^ *I*(*θ*_*j*_ ≠ 0) = |*R*| counts the number of nonzero parameters. One observation is that (2) is equivalent to (3) when reaching the optimal solution.

(3) E12y−Xθ2+λ2θ0=12y−Xθ2+λ2∑j∈R1=12y−Xθ2+λ2R.

Our *L*_0_EM methods will be derived from (3). We can rewrite (3) as the following two equations:

(4) E=12y−Xθ2+λ2∑j∈Rθj2ηj2,

(5) η=θ.

Given *η*_*j*_, (4) is a convex quadratic function and can be optimized by taking the first-order derivative:

(6) $$\begin{array}{c} \nabla E=\lambda \theta_{R}⊘\eta_{R}^{2}-X_{R}^{t}\left(\;y-X\theta \;\right)=0, \end{array}$$

where ⊘ indicates element-wise division. Rewriting (6), we have

(7) $$\begin{array}{c} \lambda \theta_{R}-\eta_{R}^{2}⊙X_{R}^{t}\left(\;y-X\theta \;\right)=0. \end{array}$$

In addition,

(8) $$\begin{array}{c} \lambda \theta_{O}-\eta_{O}^{2}⊙X_{O}^{t}\left(\;y-X\theta \;\right)=0,\;\forall \lambda >0, \end{array}$$

since *θ*_*O*_ = *η*_0_ = 0, where ⊙ is element-wise multiplication, *η*_*R*_^2^⊙*X*_*R*_^*t*^ = [*η*_*R*_^2^⊙**x**_1*R*_^*t*^,…, *η*_*R*_^2^⊙**x**_*nR*_^*t*^], and *η*_*O*_^2^⊙*X*_*O*_^*t*^ = [*η*_*O*_^2^⊙**x**_1*O*_^*t*^,…, *η*_*O*_^2^⊙**x**_*nO*_^*t*^] = 0. Let *D* = diag⁡(*η*_1_^2^,…, *η*_*m*_^2^) be a diagonal matrix with *η*_*j*_^2^s on the diagonal and combine (7) and (8) together; we have

(9) η2⊙∇Eλθ−DXty−Xθ=λθ−DXty+DXtXθ=0.

Solving (9) leads to following explicit solution:

(10) θ=DXtX+λI−1DXty,

(11) η=θ,

where (10) can be considered as the M-step of the EM algorithm maximizing negative cost function −*E* and (11) can be regarded as the E-step with *E*(*η*) = *θ*. Equations (10) and (11) together can also be treated as a fixed point iteration method in nonlinear optimization. It is guaranteed to have optimal solutions under certain conditions as shown in Theorem 1.

**Theorem 1 .**

Given an input matrix *X*, output matrix **y**, and initialized solution *θ* ^0^, the nonlinear system determined by (10) and (11) will converge to an optimal solution, when $\lambda {\left\|(DX^{t}X+\lambda I{)}^{-2}\right\|}_{\infty }{\left\|\sqrt{D}X^{t}y\right\|}_{\infty }<1/2$.

**Proof**

Equations (10) and (11) are the same as

(12) θDXtX+λI−1DXty=θ2⊙XtX+λI−1θ2⊙Xty.

First, *G*(*θ*) = (*θ* ^2^⊙*X* ^*t*^ *X* + *λI*)^−1^(*θ* ^2^⊙*X* ^*t*^)**y** is Lipschitz continuous for *θ* ∈ *R* ^*m*^, and

(13) ∇Gθ=DXtX+λI−2·θ2⊙XtX+λI2θ⊙Xty−2θ⊙XtXθ2⊙Xty=θ2⊙XtX+λI−22λθ⊙Xty=2λDXtX+λI−2DXty.

Because $\lambda {\left\|(DX^{t}X+\lambda I{)}^{-2}\right\|}_{\infty }{\left\|\sqrt{D}X^{t}y\right\|}_{\infty }<1/2$, it is clear from (13) that there is a Lipschitz constant

(14) γ∇Gθ∞=2λ2DXtX+λI−2DXty∞≤2λDXtX+λI−2∞DXty∞<2·12=1.

So the derivative for every *θ*_*j*_ is less than 1. Now, given the initial value for (10) and (11) *η* = *θ* ^0^ ∈ *R* ^*m*^, the sequence {*θ* ^*r*^} remains bounded because, ∀*i* = 1,…, *r*,

(15) θi+1−θi∞Gθi−Gθi−1∞=∇Gξθi−θi−1∞≤∇Gξ∞θi−θi−1∞where  ξ∈θi−1,θiγθi−θi−1∞≤⋯≤γiθ1−θ0∞.

And therefore

(16) θr−θ0∞∑i=0r−1θi+1−θi∞≤θ1−θ0∞∑i=0r−1γi≤θ1−θ0∞1−γ.

Now, ∀*r*, *k* ≥ 0,

(17) θr+k−θr∞Gθr+k−1−Gθr−1∞≤γθr+k−1−θr−1∞≤γGθr+k−2−Gθr−2∞≤γ2θr+k−2−θr−2∞≤⋯≤γrθk−θ0∞≤γrθ1−θ0∞1−γ.

Hence,

(18) $$\begin{array}{c} \underset{r,k\to \infty }{lim}{\left\|\;\theta^{r+k}-\theta^{r}\;\right\|}_{\infty }=0, \end{array}$$

and therefore {*θ* ^*r*^} is a Cauchy sequence that has a limit solution *θ* ^*∗*^.

Note that *G*(*θ*) is not a convex function; multiple local optimal solutions may exist. However, given initial *θ* ^0^, our algorithm always reaches the same optimal solution closest to *θ* ^0^. Assuming that there were two solutions *θ* ^*∗*^ and *θ* ^⋄^,

(19) $$\begin{array}{c} {\left\|\;\theta^{*}-\theta^{⋄}\;\right\|}_{\infty }={\left\|\;G\left(\;\theta^{*}\;\right)-G\left(\;\theta^{⋄}\;\right)\;\right\|}_{\infty }\leq \gamma {\left\|\;\theta^{*}-\theta^{⋄}\;\right\|}_{\infty }. \end{array}$$

Since *γ* < 1, (19) can only hold, if ‖*θ* ^*∗*^ − *θ* ^⋄^‖_*∞*_ = 0. That is, *θ* ^*∗*^ = *θ* ^⋄^, so the optimal solution of the EM algorithm is always the same.

Finally, the EM algorithm will be closer to the optimal solution at each step, because

(20) $$\begin{array}{c} {\left\|\;\theta^{r+1}-\theta^{*}\;\right\|}_{\infty }={\left\|\;G\left(\;\theta^{r}\;\right)-G\left(\;\theta^{*}\;\right)\;\right\|}_{\infty }\leq \gamma {\left\|\;\theta^{r}-\theta^{*}\;\right\|}_{\infty }. \end{array}$$

Theorem 1 indicates that both the regularized parameter *λ* and initial values of the parameter *θ* are important. Given an initial value *θ* ^0^, the method converges to an optimal solution as long as $\lambda {\left\|(DX^{t}X+\lambda I{)}^{-2}\right\|}_{\infty }{\left\|\sqrt{D}X^{t}y\right\|}_{\infty }<\text{1}/\text{2}\text{.}$

**Lemma 2 .**

When *λ* < ‖*DX* ^*t*^ *X*‖_*∞*_ and ${\left\|DX^{t}X\right\|}_{\infty }>\left(1/2\right){\left\|\sqrt{D}X^{t}y\right\|}_{\infty }$, the algorithm will find a nontrivial optimal solution for *θ*. More specifically, it will converge to an optimal solution, when *λ* < (1/4)‖(*X* ^*t*^ *X*)^−1^diag^2^(*X* ^*t*^ **y**)‖_*∞*_ and ‖*θ*‖_*∞*_ > (1/2)‖*X* ^*t*^ *X*‖_*∞*_^−1^‖*X* ^*t*^ **y**‖_*∞*_ for *λ* and *θ*, respectively, where diag⁡(**x**) is a diagonal matrix with *x*_*i*_ on the diagonal.

**Proof**

Since $\lambda {\left\|(DX^{t}X+\lambda I{)}^{-2}\right\|}_{\infty }{\left\|\sqrt{D}X^{t}y\right\|}_{\infty }<1/2$, we have

(21) 12λDXtX+λI−2∞DXty∞≥2λDXtX+λI∞−2DXty∞≥2λDXtX∞+λ−2DXty∞=2λDXtX∞+λDXty∞DXtX∞+λ.

Inequality (21) is satisfied, if

(22) 2λDXtX∞+λ<1,DXty∞DXtX∞+λ<1⇓λ≤DXtX∞,DXtX∞>12DXty∞.

In addition, we have

(23) DXtX∞=diag⁡θdiag⁡θtXtX∞=diag⁡DXtX+λI−1DXty·diag⁡ytXDDXtX+λI−1XtX∞≥2XtX−1diag2Xty2XtX−1XtX∞=14XtX−1diag2Xty∞,

and let

(24) D∞XtX∞DXtX∞>12D∞Xty∞≥12DXty∞.

Therefore, if we take

(25) λ<14XtX−1diag2Xty∞≤DXtX∞,θ∞=D∞>12XtX∞−1Xty∞,

the algorithm will find a nontrivial optimal solution. In particular, when *X* ^*t*^ *X* = *I*, we have

(26) λ<14diag2Xty∞=14max⁡xjty2j=1m,θ∞>12Xty∞.

Both Theorem 1 and Lemma 2 provide some useful guidance for implementing the method and choosing appropriate *λ* and *θ* ^0^. They show that the EM algorithm always converges to an optimal solution, given certain *λ* and initial solution *θ* ^0^, and the estimated value is closer to the true solution after each EM iteration. Note that there is a trivial solution *θ*_*j*_ = 0, ∀*j* = 1,…, *m*, for (10) and (11); therefore, nonzero initial *θ* ^0^ is critical for finding a nontrivial solution. Our experiences with this method indicate that initializing *θ* with the estimates from *L*_2_ penalized ridge regression will lead to quick convergence and super performance. The algorithm with such initialization usually converges under one hundred iterations and the performance is substantially better than lasso as shown in Section 3. The EM algorithm is as shown in Algorithm 1.

To deal with big data problem with *n* ≪ *m*, we also propose an efficient algorithm by solving the much smaller (*n* × *n*) matrix inverse problem similar to [19]. The algorithm is based on the following fact:

(27) $$\begin{array}{c} {\left(\;DX^{t}X+\lambda I_{m}\;\right)}^{-1}DX^{t}=DX^{t}{\left(\;XDX^{t}+\lambda I_{n}\;\right)}^{-1}. \end{array}$$

So *θ* has the analytical solution:

(28) $$\begin{array}{c} \theta =DX^{t}{\left(\;XDX^{t}+\lambda I_{n}\;\right)}^{-1}y. \end{array}$$

The dual *L*_0_EM (D*L*_0_EM) algorithm dealing with *n* ≪ *m* problem with (28) is as shown in Algorithm 2.

Apparently, while *L*_0_EM algorithm is efficient for solving the large *n* big data problem, D*L*_0_EM can handle *n* ≪ *m* problem more efficiently.

**Lemma 3 .**

Given appropriate initial *θ* ^0^, the final solution of *L*_0_EM and D*L*_0_EM algorithm is an optimal solution for *L*_0_ approximation problem that minimizes *E* = (1/2)‖**y** − *Xθ*‖^2^ + (*λ*/2) | *R*| in (3).

**Proof**

First, we show that the proposed algorithm is *L*_0_ approximation. Given a high-dimensional matrix *X*_*n*×*m*_(*n* ≪ *m*) and a threshold *γ* for the coefficient estimates, *L*_0_ rejects all the coefficient estimates below *γ* to 0 and keeps the large coefficients unchanged. This is the same as defining a binary vector *S* = [0,…, 1,…, 1]^*t*^, with the value of 0 or 1 for each feature, where *S*_*j*_ = 1, if the coefficient estimate for that feature is above the threshold *γ* and 0 otherwise. Let *S* = diag⁡(*S*) be a diagonal matrix with *S* on its diagonal; we have the selected feature matrix *X*_*S*_ = *XS*. We can build the standard models with the matrix *X*_*S*_, if we know *S* in advance. For instance, we can estimate the coefficients of a ridge regression given *X*_*S*_ and *y* with

(29) θXStXS+λI−1XSty=XStX+λI−1XtSy=SXtX+λI−1SXty,

where *X*_*S*_^*t*^ *X*_*S*_ = *SX* ^*t*^ *XS* = *SX* ^*t*^ *X* because of the special structure of matrix *S*. It is guaranteed that the estimate for feature *j* is 0 with *S*_*j*_ = 0. However, in reality, we do not know *S*. Estimating both *S* and *θ* is NP-hard problem, since we need to solve a mixed-integer optimization problem. Comparing (29) with the M-step of the primal algorithm, *θ* = (*DX* ^*t*^ *X* + *λI*_*m*_)^−1^ *DX* ^*t*^ *y*, where *D* = diag⁡(*η*_1_^2^,…, *η*_*m*_^2^); it is clear that *S* is replaced by *D* and binary *S*_*j*_ is approximated by continuous *η*_*j*_^2^ in the proposed algorithm. Therefore, The proposed algorithm is a direct *L*_0_ approximation.

Next, we show that the proposed algorithm leads to a sparse solution. Note that the penalties for *L*_0_ regularized regression in (4) are inversely proportional to the squared magnitude of the parameters. That is,

(30) $$\begin{array}{c} \lambda_{j}=\left\{\begin{array}{cc} \frac{\lambda }{2\eta_{j}^{2}} & if\;\;\eta_{j}\neq 0\\ \infty & if\;\;\eta_{j}=0, \end{array}\right. \end{array}$$

and *η* = *θ*, when *L*_0_EM or D*L*_0_EM algorithm converges. Equation (30) shows that when the true parameter *θ*_*j*_ = 0, the penalty *λ*_*j*_ goes to infinity, so ${\hat{\theta }}_{j}$ must be 0 with the proposed algorithms. In addition, when the true parameters *θ*_*j*_ ≠ 0,

(31) $$\begin{array}{c} E=\frac{1}{2}{\left\|\;y-X\theta \;\right\|}^{2}+\frac{\lambda }{2}\underset{j\in R}{\sum }\frac{\theta_{j}^{2}}{\eta_{j}^{2}}=\frac{1}{2}{\left\|\;y-X\theta \;\right\|}^{2}+\frac{\lambda }{2}\left|\;R\;\right|, \end{array}$$

because *η*_*j*_ = *θ*_*j*_, when the algorithm converges. Therefore, Lemma 3 holds. Note that our proposed methods will find a sparse solution with a large number of iterations and small *ε*, even though the solution of *L*_2_ regularized regression is not sparse. Small parameters (*θ*_*j*_s) become smaller at each iteration and will eventually go to zero (below the machine epsilon). We can also set a parameter to 0 if it is below predefined *ε* = 10*e* − 6 to speed up the convergence of the algorithm.

The proposed algorithms are similar to the iteratively reweighted least square approach for *L*_*p*_/*L*_*q*_ optimization in the literature [20, 21]. However, instead of solving *L*_*p*_ optimization problem directly, they added a small value *ε* in *θ*_*j*_^2^/(*η*_*j*_^2−*p*^ + *ε*) to handle the undefined 0/0 problem when *θ*_*j*_ = 0, leading to approximation and bias estimations. In our proposed algorithm, 0s are multiplied into the feature matrix *X* (*X*_*D*_ = *XD*). There is no undefined 0/0 problem in the proposed algorithm. Finally, similar procedures can be extended to general *L*_*p*_; *p* ∈ [0,2] without much difficulty. *L*_*p*_ based EM algorithm *L*_*p*_EM and the statistical properties of *L*_0_ penalized regression are reported in the Appendix in Supplementary Material available online at http://dx.doi.org/10.1155/2016/3456153. The proposed *L*_*p*_EM algorithm is similar to adaptive lasso [7] in that both use a weighted penalty. However, the weights in adaptive lasso are predetermined by ordinary least estimates when *n* > *m* and univariate regression coefficients when *n* < *m*, which may lead to inaccurate estimate. In contrary, our proposed *L*_*p*_EM updates the weights with an analytical solution at each iteration and automatically finds the optimal weights and estimates.

*L*_0_ *Based Local Graphical Model*. One important application of *L*_0_ regularized regression is to detect high-order correlation structures, which have numerous real-world applications including gene network analysis. Given matrix *X*, let **x**_*j*_ be the *j*th variable, and let *X*_−*j*_ be the remaining variables; we have *P*(**x**_*j*_∣*X*_−*j*_) ~ *N*(*X*_−*j*_ *θ*, *σ* ^2^), where the coefficients *θ* measure the partial correlations between **x**_*j*_ and the rest of variables. Therefore, we will minimize

(32) $$\begin{array}{c} arg\;\underset{\theta }{min}\;E\left(\;\theta \;\right)=arg\;\underset{\theta }{min}\left\{\;{\left\|\;x_{j}-X_{-j}\theta \;\right\|}^{2}+\lambda {\left\|\;\theta \;\right\|}_{0}\;\right\}. \end{array}$$

The high-order structure of *X* has been determined via a series of *L*_0_ regularized regressions for each **x**_*j*_ with the remaining variables *X*_−*j*_. The collected regression nonzero coefficients are the edges on the graph. *L*_0_ local graphical model without cross validation is much efficient computationally than *L*_1_ local graphical model. *L*_1_ local graphical model is computationally intensive, because the regularized parameter *λ* for *L*_1_ has to be determined through cross validation [22, 23]. For instance, given a matrix *X* with 100 variables, to find the optimal *λ*_opt_ from 100 candidate *λ*'s with 5-fold cross validation, 500 models need to be evaluated for each variable **x**_*j*_. Therefore a total of 500 × 100 = 50000 models have to be estimated to detect the dependency among *X* with lasso. It usually takes hours to solve this problem. However, only 100 models are required to identify the same correlation structure with *L*_0_ regularized regression and AIC or BIC, which only takes less than one minute to solve a similar problem. Notice that negative correlations between genes are difficult to confirm and seemingly less biologically relevant [24]. Most national databases are constructed with similarity (dependency) measures. It is straightforward to study only the positive dependency by simply setting *θ*  (*θ* < 0) = 0 in the EM algorithm.

*Determination of λ*. Regularized *λ* determines the sparsity of the model. The standard approach for choosing *λ* is cross validation and optimal *λ* is determined by the minimal mean squared error (MSE) of the test data ($MSE=\sum (y_{i}-\hat{y_{i}}{)}^{2}/n$). One could also adapt the stability selection (SS) approach for *λ* determination [25, 26]. It chooses smallest *λ* that minimizes the inconsistencies in number of nonzero parameters with cross validation. We first calculate the mean and standard deviation (SD) of the number of nonzero parameters for each *λ* and then find smallest *λ* with SD = 0, where SD = 0 indicates that all models in *k*-fold cross validation have the same number of nonzero estimates. Our experiences indicate that larger *λ* chosen from both minimal MSE and stability selection (*λ* = max⁡{*λ*_MSE_, *λ*_SS_}) has the best performance. Choosing optimal *λ* from cross validation is computationally intensive and time-consuming. Fortunately, unlike lasso, identifying optimal *λ* for *L*_0_ does not require using cross validation. Optimal *λ*_opt_ can be determined by variable selection criteria. Optimal *λ*_opt_ can be directly picked using AIC, BIC, or RIC criteria with *λ*_opt_ = 2, log⁡*n*, or 2log⁡*m*, respectively. Each of these criteria is known to be optimal under certain conditions. This is a huge advantage of *L*_0_, especially for big data problems. In general, we recommend to use BIC as information criteria for high-dimensional problem (*n* ≪ *p*) and to use AIC when *n* > *p*.

## 3. Results

### 3.1. Simulation Study Application

To evaluate the performance of *L*_0_ and *L*_1_ regulation, we assume a linear model **y** = *Xθ* + *ε*, where the input matrix *X* is from Gaussian distribution with mean *μ* = 0 and different covariance structures Σ, where Σ(*i*, *j*) = *r* ^|*i*−*j*|^ with *r* = 0,0.3,0.6,0.8, respectively. The true model is **y** = 2**x**_1_ − 3**x**_2_ + 4**x**_5_ + *ε* with *ε* ~ *N*(0,1). Therefore, only three features are associated with output **y**, and the rest of *θ*_*i*_'s are zero. In our first simulation, we first compare *L*_0_ and *L*_1_ regularized regressions with a relatively small number of features *m* = 50 and a sample size of *n* = 100. Fivefold cross validation is used to determine optimal *λ* and compare the models performances. We seek to fit the regularized regression models over a range of regularization parameters *λ*. Each *λ* is chosen from *λ*_min_ = 1*e* − 4 to *λ*_max_ with 100 equally log-spaced intervals, where *λ*_max_ = max⁡{*X* ^*t*^ **y**} for *L*_1_ and max⁡{(**x**_*j*_^*t*^ **y**)^2^/4**x**_*j*_^*t*^ **x**_*j*_} for *L*_0_. Lasso function in the statistics toolbox of MATLAB (http://www.mathworks.com/) is used for comparison. Cross validation with MSE is implemented nicely in the toolbox. The computational results are reported in Table 1. Table 1 shows that *L*_0_ outperforms lasso in all categories by a substantial margin, when using the popular test MSE measure for model selection. In particular, the number of variables selected by *L*_0_ are close to 3, the true number of nonzero variables, while lasso selected more than 11 features on average with different correlation structures (*r* = 0,0.3,0.6,0.8). The test MSEs and bias both increase with the growth of correlation among features for both *L*_0_ and lasso, but the test MSE and bias of *L*_0_ are substantially lower than these of lasso. The maximal MSE of *L*_0_ is 1.06, while the smallest MSE of *L*_1_ is 1.19, and the largest bias of *L*_0_ is 0.28, while the smallest bias of lasso is 0.38. In addition (results are not shown in Table 1), *L*_0_ correctly identifies the true model 81, 74, 81, and 82 times for *r* = 0,0.3,0.6 and 0.8, respectively, over 100 simulations, while lasso never chooses the correct model. Therefore, compared to *L*_0_ regularized regression, lasso selects more features than necessary and has larger bias in parameter estimation. Even though it is possible to get a correct model with lasso using larger *λ*, the estimated parameters will have a bigger bias and worse predicted MSE.

The same parameter setting is used for our second simulation, but the regularized parameter *λ* is determined by larger *λ* from both minimal MSE and stability selection (*λ* = max⁡{*λ*_MSE_, *λ*_SS_}). The computational results are reported in Table 2. Table 2 shows that the average number of associated features is much closer to 3 with slightly larger test MSEs. The maximal average number of features is 3.1 with *r* = 0.6, reduced from 3.49 with the test MSE only. In fact, with this combined model selection criteria and 100 simulations, *L*_0_EM identified the true model with three nonzero parameters 95, 95, 95, and 97 times, respectively (not shown in the table), while lasso did not choose any correct models. The average bias of the estimates with *L*_0_EM is also reduced. These indicate that the combination of test MSE and stability selection in cross validation leads to better model selection results than MSE alone with *L*_0_EM. However, the computational results did not improve much with lasso. Over 13 features on average were selected under different correlation structures, suggesting that lasso inclines to select more spurious features than necessary. A much more conservative criterion with larger *λ* is required to select the right number of features, which will induce larger MSE and bias and deteriorate the prediction performance.

**Figure 1:**
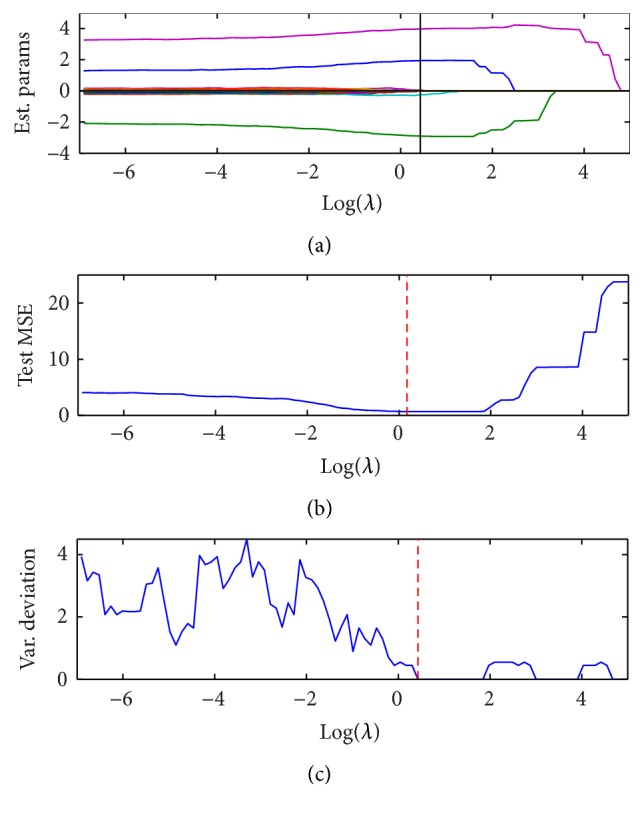
Regularized path for *L*_0_ penalized regression with *n* = 100, *m* = 1000, and *r* = 0.3.

*Simulation with High-Dimensional Data*. Our third simulation deals with high-dimensional data with the number of samples *n* = 100 and the number of features *m* = 1000. The correlation structure is set to *r* = 0,0.3,0.6, and the same model **y** = 2**x**_1_ − 3**x**_2_ + 4**x**_5_ + *ε* was used for evaluating model performance. Besides *L*_1_, *L*_0_ was also compared with two other cutting-edge regulations including SCAD and MC+, implemented in SparseReg package [27]. The simulation was repeated 100 times. The computational results are reported in Table 3. Table 3 shows that *L*_0_ outperforms lasso by a large margin when correlations among features are low. When there is no correlation among features, 100 out of 100 simulations identify the true model with *L*_0_, and 78 out of 100 simulations choose the correct model when *r* = 0.3, while lasso, SCAD, and MC+ choose more features than necessary and no true model was found under any correlation setting. However, when correlations among features are large with *r* = 0.6, the results are mixed. *L*_0_ can still identify 23 out of 100 correct models, but the test MSE and bias of the parameter estimate of *L*_0_ are slightly large than those of lasso, MC+, and SCAD. MC+ has the second best performance with less bias and smaller test MSE but chooses more features than necessary. In addition, we notice that *L*_0_ is a more sparse model when correlation increases, indicating that *L*_0_ tends to choose independent features. One reason for selecting more features with SCAD and MC+ may be that we only tuned the parameter *λ* and fixed *γ* = 3.7 and *γ* = 1 for SCAD and MC+, respectively. A regularization path of *L*_0_ regression is shown in Figure 1. As shown in Figure 1(a), the three associated features first increase their values when *λ* goes larger and then go to zero when *λ* becomes extremely big, while the rest of the irrelevant features all go to zero when *λ* increases. Unlike lasso, which shrinks all parameters uniformly, *L*_0_ will only force the estimates of irrelevant features to go to zero, while keeping the estimates of relevant features to their true value. This is the well-known oracle property of *L*_0_. The oracle property means that the penalized estimator is asymptotically equivalent to the oracle estimator obtained only with signal variables without penalization. For this specific simulation, the three parameters $\left[\hat{\theta_{1}},\hat{\theta_{2}},\hat{\theta_{5}}]=[1.85,-2.94,4.0\right]$, very close to their true values [2, −3,4]. Figures 1(b) and 1(c) are the test MSE and the standard deviation of the number of nonzero variables. Optimal *λ* is chosen from larger *λ* with minimal test MSE and stability selection as shown in the vertical lines of Figure 1.

*L*_0_ *Regularized Regression without Cross Validation*. Choosing the optimal parameter *λ*_opt_ with cross validation is time-consuming, especially with big data. As we mentioned previously, optimal *λ* can be picked from theory instead of cross validation. Since we are dealing with *n* ≪ *m* big data problem, RIC with *λ*_opt_ = 2log⁡*m* tends to penalize the parameters too much. So computational results with AIC and BIC without cross validation are reported in Table 4. Table 4 shows that *L*_0_ regularized regression with AIC and BIC performs very well when compared with the results from computationally intensive cross validation in Table 3. Without correlation, BIC identifies the true model (100%), which is the same as cross validation in Table 3 and better than AIC's 78%. The bias of BIC (0.16) is only slightly higher than that of cross validation (0.14) but lower than that of AIC (0.19). Even though MSE^*∗*^'s with AIC and BIC are in-sample mean squared errors, which are not comparable to the test MSE with cross validation, larger MSE^*∗*^ with BIC indicates that BIC is a more stringent criterion than AIC and selects less variables. With mild correlation (*r* = 0.3) and some sacrifices in bias and MSE^*∗*^, BIC performs better than AIC in variable selection, since the average number of features selected is exactly 3 and 94% of the simulations recognize the true model, while AIC chooses more features (3.72) than necessary and only 73% of the simulations are right on targets. Cross validation is the most tight measure with 2.9 features on average and 75% of the simulations finding the correct model. When the correlations among the variables are high (*r* = 0.6), the results are mixed. Both BIC and AIC correctly identify more than half of the true models, while cross validation only recognizes 25% (5/20) of the model correctly. Therefore, compared with the computationally intensive cross validation, both BIC and AIC perform reasonably well. The performance of BIC is similar to cross validation with less computational time. In addition, we have suggested to use the result of ridge regression as the initial value for the proposed algorithms. However, the proposed algorithm is quite stable with different initializations. With *n* = 100, *p* = 200, *r* = 0.3, and 100 times of randomized initializations, the estimates of three nonzero parameters are [*β*_1_, *β*_2_, *β*_5_] = [2.05 ± 0.08, −2.89 ± 0.08,4.01 ± 0.09] with BIC criteria.

*Simulations for Graphical Models*. We simulate two network structures similar to those in Zhang and Mallick [28]: (i) band 1 network, where Σ is a covariance matrix with *σ*_*ij*_ = 0.6^|*i*−*j*|^, so *A* = Σ^−1^ has a band 1 network structure, and (ii) a more difficult problem for a band 2 network with weaker correlations, where *A* = −Σ^−1^ with

(33) $$\begin{array}{c} a_{ij}=\left\{\begin{array}{cc} 0.25 & if\;\;\left|i-j\right|=1\\ 0.4 & if\;\;\left|i-j\right|=2\\ 0 & otherwise. \end{array}\right. \end{array}$$

The sample sizes are *n* = 50, 100, and 200, respectively, and the number of variables is *m* = 100. *L*_0_ regularized regression with AIC and BIC is used to detect the network (correlation) structure. The consistency between the true and predicted structures is measured by the area under the ROC curve (AUC), false discovery (positive) rate (FDR/FPR), and false negative rate (FNR) of edges. The computational results are shown in Table 5. Table 5 shows that both AIC and BIC performed well. Both achieved at least 0.90 AUC for band 1 network and 0.8 AUC for band 2 network with different sample sizes. AIC performed slightly better than BIC, especially for band 2 network with weak correlations and small sample sizes. This is reasonable because BIC is a heavier penalty and forces most of the weaker correlations with *a*_*ij*_ = 0.25 to 0. In addition, BIC has slightly larger AUCs for band 1 network with strong correlation *r* = 0.6 and larger sample size (*n* = 100, 200). One interesting observation is that FDRs of both AIC and BIC are well controlled. Maximal FDRs of AIC for bands 1 and 2 networks are 0.29% and 0.2%, while maximal FDRs of BIC are only 0.1%, and 0.03%, respectively. Controlling false discovery rates is crucial for identifying true associations with high-dimensional data in bioinformatics. In general, AUC increases and both FDR and FNR decrease, as the sample sizes become larger, except for band 2 network with BIC. The performance of BIC is not necessarily better with a larger sample size, since the penalty *λ* increases with the sample size. *L*_1_ graphical model was also used for comparison purpose [29, 30]. *L*_1_ graphical model performed equally well as AIC and BIC with band 1 network but was the worst with the more difficult band 2 network. More interestingly, *L*_1_ had the largest FDR, indicating that it selects more features than necessary.

### 3.2. Application to Real Ovarian Cancer Data

**Figure 2:**
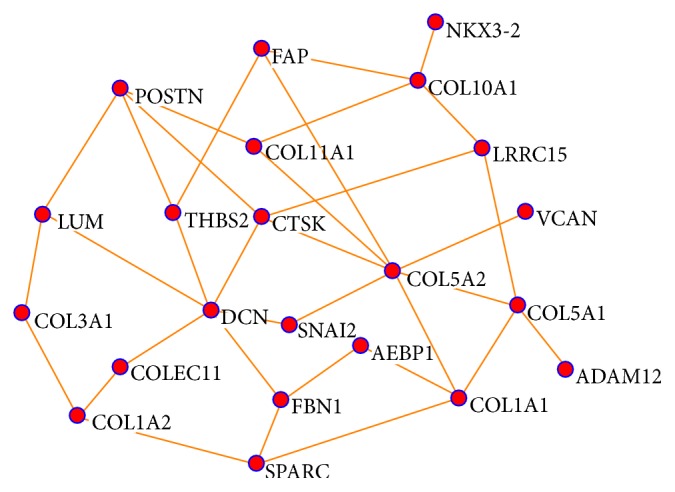
Subnetwork constructed with *L*_0_ penalized regression, multisource gene expression profiling, and BIC.

**Figure 3:**
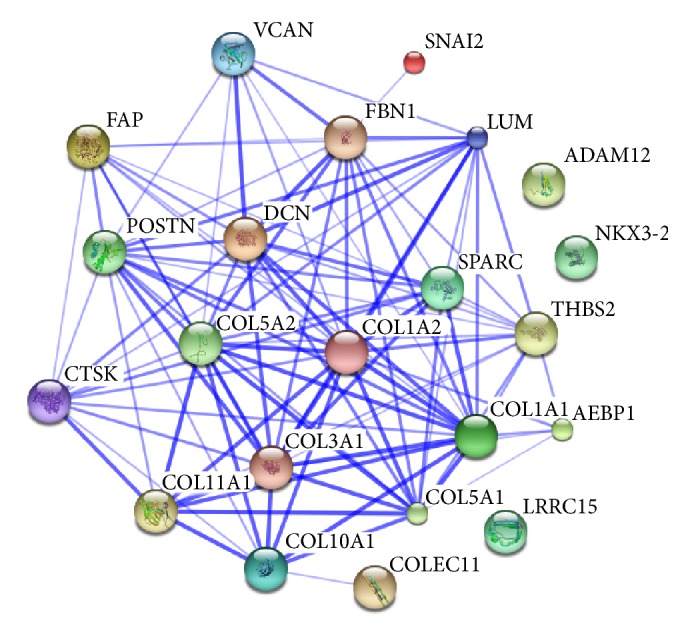
Known and predicted protein-protein interactions with the 22 genes on the subnetwork of Figure 2, where nodes represent proteins (genes) and edges indicate the direct (physical) and indirect (functional) associations. Stronger associations are represented by thicker lines.

The purpose of this application is to identify subnetworks and study the biological mechanisms of potential prognostic biomarkers for ovarian cancer with multisource gene expression data. The ovarian cancer data was downloaded from the KMplot website (http://www.kmplot.com/ovar/) [31]. They originally got the data from searching Gene Expression Omnibus (GEO; http://www.ncbi.nlm.nih.gov/geo/) and The Cancer Genome Atlas (TCGA; http://cancergenome.nih.gov/) with multiple platforms. All collected datasets have raw gene expression data, survival information, and at least 20 patients available. They merged the datasets across different platforms carefully. The final data has 1287 patients samples and 22277 probe sets representing 13435 common genes. We identified 112 top genes that are associated with patient survival times using univariate Cox regression. We constructed a coexpression network from the 112 genes with *L*_0_ regularized regression and identified biologically meaningful subnetworks (modules) associated with patient survival. Network is constructed with positive correlation only and BIC. The computational time for constructing such network is less than 2 seconds. One survival associated subnetwork we identified is given in Figure 2. The 22 genes on the subnetwork were then uploaded onto STRING (http://string-db.org/). STRING is an online database for exploring known and predicted protein-protein interactions (PPI). The interactions include direct (physical) and indirect (functional) associations. The predicted methods for PPI implemented in STRING include text mining, national databases, experiments, coexpression, cooccurrence, gene fusion, and neighborhood on the chromosome. The PPI networks for the 22 genes are presented in Figure 3. Comparing Figures 3 and 2, we conclude that the 22 identified genes on the subnetwork of Figure 2 are functioning together and have enriched important biological interactions and associations. Nineteen out of 22 genes on the survival associated subnetwork also have interactions on the known and predicted PPI network, except for genes LRRC15, ADAM12, and NKX3-2. Even though they are not completely identical, many interactions on our subnetwork can also be verified on the PPI interaction network of Figure 3. For instance, collagen COL5A2 is the most important gene with the largest number of degrees (7) on our subnetwork. Six out of 7 genes that link to COL5A2 also have direct edges on the PPI network. Those direct connected genes (proteins) include FAP, CTSK, VCAN, COL1A1, COL5A1, and COL11A1. The remaining gene SNAI2 was indirectly linked to COL5A2 through FBN1 on the PPI network. In addition, one of the other important genes with the degree of the node (6) is Decorin (DCN). Four out of 6 genes directly connected to DCN on our subnetwork were confirmed on the PPI network, including FBN1, CTSK, LUM, and THBS2. The remaining two genes (SNAI2 and COLEC11) are indirectly connected to DCN on the PPI network. As indicated on Figure 2, the remaining 5 important genes with degree of node 4 are POSTN, CTSK, COL1A1, COL5A1, and COL10A1, and 8 genes with degree of node 3 are FBN1, LUM, LRRC15, COL11A1, THBS2, SPARC, COL1A2, and FAP, respectively. Furthermore, those 22 genes are involved in the biological process of GO terms, including extracellular matrix organization and disassembly and collagen catabolic, fibril, and metabolic processes. They are also involved in several important KEGG pathways including ECM-receptor interaction, protein digestion and absorption, amoebiasis, focal adhesion, and TGF-*β* signaling pathways. Finally, a large proportion of the 22 genes are known to be associated with poor overall survival (OS) in ovarian cancer. For instance, VCAN and POSTN were demonstrated* in vitro* to be involved in ovarian cancer invasion induced by TGF-*β* signaling [32], and COL11A1 was shown to increase continuously during ovarian cancer progression and to be highly overexpressed in recurrent metastases. Knockdown of COL11A1 reduces migration, invasion, and tumor progression in mice [33]. Other genes such as FAP, CTSK, FBN1, THBS2, SPARC, and COL1A1 are also known to be ovarian cancer associated [34–39]. Those genes contribute to cell migration and the progression of tumors and may be potential therapeutic targets for ovarian cancer, indicating that the proposed method can be used to construct biologically important networks efficiently.

## 4. Discussion

We proposed efficient EM algorithms for variable selection with *L*_0_ regularized regression. The proposed algorithms find the optimal solutions of *L*_0_ through solving a sequence of *L*_2_ based ridge regressions. Given an initial solution, the algorithm will be guaranteed to converge to a unique solution under mild conditions, and the EM algorithm will be closer to the optimal solution after each iteration. Asymptotic properties, namely, consistency and oracle properties for exact *L*_0_, are established under mild conditions. Our method applies to fixed, diverging, and ultra-high-dimensional problems with ten or hundred thousands of features. We compare the performance of *L*_0_ regularized regression and lasso with simulated low- and high-dimensional data. *L*_0_ regularized regression outperforms lasso, SCAD, and MC+ by a substantial margin under different correlation structures. Unlike lasso, which selects more features than necessary, *L*_0_ regularized regression chooses the true model with high accuracy, less bias, and smaller test MSE, especially when the correlation is weak. Cross validation with the computation of the entire regularization path is computationally intensive and time-consuming. Fortunately *L*_0_ regularized regression does not require it. Optimal *λ*_opt_ can be directly determined from AIC, BIC, and RIC. Those criteria are optimal under appropriate conditions. We demonstrate that both AIC and BIC performed well when compared to cross validation. Therefore, there is a big computational advantage of *L*_0_, especially with big data. In addition, we demonstrate that *L*_0_ regularized regression controls the false discovery (positive) rate (FDR) well with both AIC and BIC with the simulation of graphical models. The FDR is very low under different sample sizes with both AIC and BIC. Controlling FDR is crucial for biomarker discovery and computational biology, because further verifying the candidate biomarkers is time-consuming and costly. We applied our proposed method to construct a network for ovarian cancer from multisource gene expression data and identified a subnetwork that is important both biologically and clinically. We demonstrated that we can identify biologically important genes and pathways efficiently. Even though we demonstrated our method with gene expression data, the proposed method can be used for RNA-seq and metagenomic data, given that the data are appropriately normalized. Finally, because of the nonconvexity of *L*_0_ regularized regression, there are multiple local optimal solutions for *θ*_*j*_ including a trivial solution *θ*_*j*_ = 0, ∀*j* = 1,…, *m*, as shown in (28). However, the nontrivial solution can be found efficiently as long as all parameters were initialized with nonzero values. We recommend the solution of ridge regression as an initial solution for the proposed algorithms.

## Supplementary Material

LpEM Algorthm and Statistical Properties.

## Figures and Tables

**Algorithm 1:**
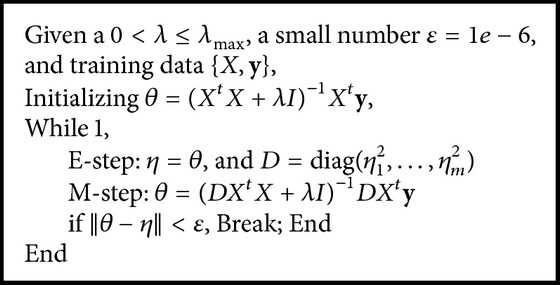
*L*_0_EM algorithm.

**Algorithm 2:**
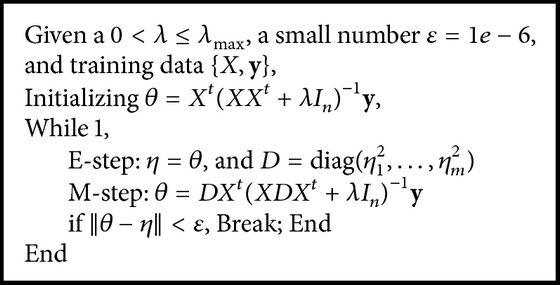
D*L*_0_EM algorithm.

**Table 1:** Performance measures for *L*_0_ and *L*_1_ regularized regression over 100 simulations, where values in the parentheses are the standard deviations. # SF: number of average selected features; MSE: average mean squared error; $\left\|\hat{\theta }-\theta \right\|$: average absolute bias when comparing true and estimated parameters.

*r*	*L*_0_			*L*_1_		
	# SF	MSE	$\left\|\hat{\theta }-\theta \right\|$	# SF	MSE	$\left\|\hat{\theta }-\theta \right\|$
0	3.39 (±1.1)	1.01 (±0.14)	0.206 (±0.12)	14.5 (±3.45)	1.19 (±0.19)	0.38 (±0.1)
0.3	3.37 (±0.9)	1.02 (±0.16)	0.23 (±0.12)	14.5 (±2.91)	1.21 (±0.19)	0.41 (±0.19)
0.6	3.49 (±1.7)	1.02 (±0.23)	0.23 (±0.16)	13.5 (±3.0)	1.26 (±0.2)	0.54 (±0.15)
0.8	3.32 (±0.9)	1.06 (±0.15)	0.28 (±0.21)	11.7 (±2.69)	1.3 (±0.21)	0.89 (±0.25)

**Table 2:** Performance measures for *L*_0_ and *L*_1_ regularized regression with *λ* = max⁡{*λ*_MSE_, *λ*_SS_} over 100 simulations, where values in the parenthesis are the standard deviations. # SF: number of average selected features; MSE: average mean squared error; $\left\|\hat{\theta }-\theta \right\|$: average absolute bias when comparing true and estimated parameters.

*r*	*L*_0_			*L*_1_		
	# SF	MSE	$\left\|\hat{\theta }-\theta \right\|$	#SF	MSE	$\left\|\hat{\theta }-\theta \right\|$
0	3.09 (±0.53)	1.04 (±0.15)	0.18 (±0.11)	13.3 (±4.56)	1.21 (±0.17)	0.39 (±0.1)
0.3	3.08 (±0.54)	1.04 (±0.15)	0.17 (±0.07)	14.5 (±4.20)	1.22 (±0.17)	0.42 (±0.19)
0.6	3.10 (±0.46)	1.07 (±0.17)	0.21 (±0.10)	13.8 (±5.4)	1.27 (±0.47)	0.57 (±0.25)
0.8	3.02 (±0.14)	1.04 (±0.14)	0.26 (±0.13)	13.4 (±4.91)	1.25 (±0.21)	0.74 (±0.25)

**Table 3:** Performance measures for *L*_0_, *L*_1_, SCAD, and MC+ regularized regressions with cross validation and *λ* = Max⁡⁡{*λ*_MSE_, *λ*_SS_} over 100 simulations and the sample size of *n* = 100, and *m* = 1000, where values in the parenthesis are the standard deviations. # SF: number of average selected features; MSE: average mean squared error; $\left\|\hat{\theta }-\theta \right\|$: average absolute bias when comparing true and estimated parameters.

	Measures	*r* = 0	*r* = 0.3	*r* = 0.6
*L*_0_	# SF	3 (±0)	2.9 (±0.47)	2 (±0.73)
	$\left\|\hat{\theta }-\theta \right\|$	0.14 (±0.09)	0.39 (±0.63)	1.69 (±1.25)
	Test MSE	1.14 (±0.34)	1.59 (±1.3)	2.8 (±1.72)
	# true model	100/100	78/100	23/100

*L*_1_	# SF	24 (±18.4)	31.3 (±20.7)	36.7 (±16.5)
	$\left\|\hat{\theta }-\theta \right\|$	0.57 (±0.11)	0.73 (±0.13)	1.14 (±0.25)
	Test MSE	1.50 (±0.25)	1.63 (±0.29)	1.92 (±0.41)
	# true model	0/100	0/100	0/100

SCAD	# SF	106.8 (±110.6)	73 (±111)	56.2 (±62.4)
	$\left\|\hat{\theta }-\theta \right\|$	0.62 (±0.13)	0.72 (±0.14)	1.13 (±0.26)
	Test MSE	1.32 (±0.27)	1.54 (±0.27)	2.04 (±0.51)
	# true model	0/100	0/100	0/100

MC+	# SF	60.3 (±38.6)	70.5 (±26.0)	78.73 (±16.5)
	$\left\|\hat{\theta }-\theta \right\|$	0.56 (±0.14)	0.66 (±0.12)	0.78 (±0.17)
	Test MSE	1.25 (±0.21)	1.31 (±0.27)	1.46 (±0.27)
	# true model	0/100	0/100	0/100

**Table 4:** Performance measures for *L*_0_ regularized regression with AIC and BIC over 100 simulations with *n* = 100, and *m* = 1000, where values in the parenthesis are the standard deviations. # SF: number of average selected features; MSE^*∗*^: in-sample average mean squared error; $\left\|\hat{\theta }-\theta \right\|$: average absolute bias when comparing true and estimated parameters.

	Measures	*r* = 0	*r* = 0.3	*r* = 0.6
AIC	# SF	3.26 (±0.54)	3.72 (±1.94)	4.8 (±2.77)
	$\left\|\hat{\theta }-\theta \right\|$	0.19 (±0.09)	0.36 (±0.58)	1.02 (±1.2)
	MSE^*∗*^	0.96 (±0.14)	1.02 (±0.31)	1.27 (±0.51)
	# true model	78/100	73/100	59/100

BIC	# SF	3.0 (±0.0)	3.0 (±0.38)	2.89 (±0.80)
	$\left\|\hat{\theta }-\theta \right\|$	0.16 (±0.08)	0.45 (±0.69)	1.80 (±1.20)
	MSE^*∗*^	0.97 (±0.15)	1.29 (±0.81)	2.48 (±1.17)
	# true model	100/100	94/100	53/100

**Table 5:** Performance measures for *L*_0_ regularized regression for graphical structure detection over 100 simulations, where values in the parenthesis are the standard deviations.

	Band 1			Band 2		
AIC	AUC	FDR (%)	FNR (%)	AUC	FDR (%)	FNR (%)
*n* = 50	.95 (±.01)	.29 (±.08)	9.4 (±2.6)	.82 (±.01)	.10 (±.05)	36.7 (±1.5)
100	.99 (±.005)	.20 (±.06)	1.2 (±1.1)	.84 (±.01)	.11 (±.04)	32.7 (±1.9)
200	.999 (±.0003)	.20 (±.05)	0 (±0)	.93 (±.01)	.11 (±.04)	14.2 (±2.4)

BIC	AUC	FPR (%)	FNR (%)	AUC	FPR (%)	FNR (%)

*n* = 50	.90 (±.02)	.10 (±.05)	20 (±3.6)	.803 (±.008)	.02 (±.02)	39.3 (±1.5)
100	.991 (±.007)	.03 (±.03)	1.8 (±1.3)	.83 (±.01)	.03 (±.02)	34.9 (±1.6)
200	.9999 (±.0005)	.01 (±.01)	.01 (±.10)	.82 (±.01)	.03 (±.02)	36.7 (±1.8)

*L*_1_	AUC	FPR (%)	FNR (%)	AUC	FPR (%)	FNR (%)

*n* = 50	.91 (±.03)	3.5 (±.05)	11 (±3.6)	0.77 (±.01)	5.3 (±.07)	40.9 (±.62)
100	.99 (±.003)	1.52 (±.22)	.33 (±.67)	0.78 (±.007)	7.1 (±1.4)	36.3 (±1.1)
200	.99 (±.003)	1.21 (±.07)	.45 (±.53)	0.79 (±.01)	8.1 (±.57)	34.0 (±1.4)
