# Supplementary material for: Efficient Regularized Regression with L 0 Penalty for Variable Selection and Network Construction
Source: Comput Math Methods Med. 2016 Oct 24;2016:3456153. doi: 10.1155/2016/3456153 (PMC5098106; doi:10.1155/2016/3456153)
Supplement: Supplementary file 1 — LpEM Algorthm and Statistical Properties. [file 3456153.f1.pdf]

Appendix: L_pEM and Statistical Properties

L_pEM Algorithm:

The proposed approach for L_0 regularized regression method can be extended to solve a general L_p $p \in (0, 2]$ problem naturally, which includes the well known elastic net with $p \in [1, 2]$ (Zou & Zhang 2009) and the combination of L_1 and L_0 with $p \in (0, 1]$ (Liu & Wu, 2007). Mathematically, the general L_p problem can be defined as:

$$E = \frac{1}{2}||\mathbf{y} - X\theta||^2 + \frac{\lambda}{2}\sum_{j=1}^{m} |\theta|^p,$$

which is equivalent to

$$E = \frac{1}{2}||\mathbf{y} - X\theta||^2 + \frac{\lambda}{2}\sum_{j \in m} \frac{\theta_j^2}{\eta_j^{2-p}}$$
$$\eta = \theta.$$

let $D_p = diag(\eta_1^{2-p}, \dots, \eta_m^{2-p})$, similar ideas in the manuscript can be used to get the following equation for the general L_pEM method:

$$\eta^{2-p} \odot \frac{\partial E}{\partial \theta} = \lambda\theta - D_p X^t(\mathbf{y} - X\theta) = \lambda\theta - D_p X^t(\mathbf{y} - X\theta) = 0.$$

Solving above equation, we have the following explicit solution.

$$\theta = (D_p X^t X + \lambda I)^{-1} D_p X^t \mathbf{y}$$
$$\eta = \theta,$$

The general L_pEM algorithm is as follows:

L_pEM Algorithm:

Given a $0 < \lambda \le \lambda_{\max}$,and $p \in [0, 2]$, $\epsilon = 1e - 6$ and $\varepsilon = 1e - 6$,
and training data $\{X, \mathbf{y}\}$,
Initializing $\theta = (X^t X + \lambda I)^{-1} X^t \mathbf{y}$,
While 1,
  E-step: $\eta = \theta$, and $D_p = diag(\eta_1^{2-p}, \dots, \eta_m^{2-p})$
  M-step: $\theta = (D_p X^t X + \lambda I)^{-1} D_p X^t \mathbf{y}$
  if $||\theta - \eta|| < \varepsilon$, Break; End
End

Statistical Properties for Exact L_0 Regularized Regression:

Consistency and Oracle Property: Let θ_0 be the true parameter value. The following conditions will be used later for theoretical properties of the L_0-regularized estimator of θ_0.
CONDITIONS

(C1) $ln(m) = o(n)$ as $n \to \infty$.

(C2) There exists a constant $K > 0$ such that $\lambda_{max}(\frac{X^t X}{n}) \le K < \infty$ for large n, where for any matrix B, $\lambda_{max}(B)$ denotes the largest eigenvalue of B.

(C3) $\frac{\max_j ||\mathbf{x}_j||}{\sqrt{n}} = O(\sqrt{ln(mn)})$ or $O(1)$ as $n, m \to \infty$.

(C4) There exists a constant $c > 0$ such that $\frac{\min_j ||\mathbf{x}_j||^2}{n} \geq c > 0$ for large n, m.

(C5) $\mu(X) \equiv \max_{1\leq i<j\leq m} \frac{|\mathbf{x}_i^t \mathbf{x}_j|}{||\mathbf{x}_i||\cdot||\mathbf{x}_j||} = O(\sqrt{\frac{ln(m)}{n}})$.

(C6) $||\theta||_0 = O(1)$.

The above conditions are very mild. Condition (C1) trivially holds for $m \leq n$. In particular, (C1) is satisfied even for ultra-high dimensional case such as $m = exp(n^{\alpha})$ for $0 < \alpha < 1$. (C2) is a standard condition for linear regression. Chi (2013, Section 3.2) gives examples satisfying(C3)-(C4). For example, (C3) and (C4) trivially hold if $||\mathbf{x}_i|| = \sqrt{n}$ for all $j = 1, \ldots, m$. (C5) is referred to as the coherence condition under which the covariates are not highly colinear; see Bunea et al. (2007), Candes and Plan (2009), and Chi (2013). (C6) implies that the model is sparse.

The following theorem is a direct consequence of Chi (2013).

Theorem 1 (***Consistency***) *Assume that conditions (C1)-(C6) hold. Let $n(\nu) = (1-\nu)[1 + 1/\mu(X)]$ for some $0 < \nu < 1$. For any $0 < q < \frac{1}{2}$, let $\lambda = \frac{3ln(m/q)}{\nu[1+\mu(X)]} \frac{\max_j ||\mathbf{x}_j||^2}{\min_j ||\mathbf{x}_j||^2}$, and*

$$\hat{\theta} = arg \min_{||\theta||_0 \leq n(\nu)} E_n(\theta).$$

Then, with probability tending to 1,

$$||\hat{\theta} - \theta_0|| = O_p(\sqrt{\frac{\ln(nm)}{n}}) \tag{1}$$

Proof Note that the normal linear model in this paper is a special case of the exponential model of Chi (2009): $p_t(y) = \exp(ty - \Lambda(t))$ with $t = \frac{\mathbf{x}_i^t \theta}{\sigma^2}$ and $\Lambda(t) = \frac{\sigma^2 t^2}{2}$. Then, (**??**) follows immediately from Theorem 3.1 of Chi (2009).

Model Recovery: Next we show that with large probability L_0-regularized regression recovers the true model under mild conditions.

Theorem 2 (***Oracle Property***) *Assume that conditions (C1)-(C6) hold. Let $A = \{1 \leq j \leq m : \theta_{0j} \neq 0\}$, and $A^c = \{1, 2, \ldots, m\} \backslash A$. Then, the minimizer $\hat{\theta}$ in Theorem* **??** *must satisfy $\hat{\theta}_j = 0$ for $j \in A^c$ with probability tending to 1 as n goes to ∞.*

Proof Let $\alpha_n = \sqrt{\frac{\ln(nm)}{n}}$. For any θ such that $||\theta - \theta_0|| < C\alpha_n$ for some constant $C > 0$ and $\sum_{j \in A^c} I(\theta_j \neq 0) \geq 1$, let

$$\tilde{\theta}_j = \begin{cases} \theta_j \text{ if } j \in A \\ 0 \text{ if } j \in A^c \end{cases}$$

Then,

$$
\begin{aligned}
&E_n(\theta) - E_n(\tilde{\theta}) \\
&= \frac{1}{2n}(\theta-\tilde{\theta})^T X^T X(\theta-\tilde{\theta}) - \frac{1}{n}(\theta-\tilde{\theta})^T X^T(y - X\tilde{\theta}) + \frac{\lambda}{2}(||\theta||_0 - ||\tilde{\theta}||_0) \\
&= \frac{1}{2n}(\theta-\tilde{\theta})^T X^T X(\theta-\tilde{\theta}) - \frac{1}{n}(\theta-\tilde{\theta})^T X^T(X\theta_0 + \epsilon - X\tilde{\theta}) + \frac{\lambda}{2}(||\theta||_0 - ||\tilde{\theta}||_0) \\
&= \frac{1}{2}(\theta-\tilde{\theta})^T \left(\frac{X^T X}{n}\right)(\theta-\tilde{\theta}) - (\theta-\tilde{\theta})^T\left(\frac{X^T X}{n}\right)(\theta_0-\tilde{\theta}) + \\
&+ \frac{1}{\sqrt{n}}(\theta-\tilde{\theta})^T \cdot \frac{1}{\sqrt{n}}X^T\epsilon + \frac{\lambda}{2}(||\theta||_0 - ||\tilde{\theta}||_0) \\
&= I_1 + I_2 + I_3 + I_4
\end{aligned}
$$

Because $||\tilde{\theta} - \theta_0|| \leq ||\theta - \theta_0||$, we have $\theta - \tilde{\theta} = O(\alpha_n)$. Thus, $I_1 = O(\alpha_n^2)$ and $I_2 = O(\alpha_n^2)$. Moreover,

$$
\left|\left|\frac{1}{\sqrt{n}}\epsilon^t X\right|\right| = O_p(\sqrt{k\sigma^2}), \qquad \text{as } n \to \infty
$$

where $k = rank(X) \leq n$. Hence,

$$
|I_3| \leq \frac{1}{\sqrt{n}}||\theta - \tilde{\theta}|| \cdot \left|\left|\frac{1}{\sqrt{n}}X^T\epsilon\right|\right| = O(\alpha_n)\cdot O_p(\sqrt{k/n}) = O_p(\alpha_n).
$$

Furthermore,

$$
\begin{aligned}
I_4 &= \frac{\lambda}{2}(||\theta||_0 - ||\tilde{\theta}||_0) \\
&= \frac{\lambda}{2}\sum_{j=1}^{m}[I(\theta_j \neq 0) - I(\tilde{\theta}_j \neq 0)] \\
&= \frac{\lambda}{2}\left[\sum_{j\in A} 0\right] + \frac{\lambda}{2}\sum_{j\in A^c}[I(\theta_j \neq 0) - 0] \\
&= \frac{\lambda}{2}\sum_{j\in A^c} I(\theta_j \neq 0) \geq \frac{\lambda}{2}\cdot 1 > 0.
\end{aligned}
$$

By conditions (C3)-C(5), $\lambda = O(ln(m)\cdot ln(nm))$. Therefore, the first three terms I_1, I_2 and I_3 are dominated by λ in probability as $n \to \infty$. Therefore, with probability tending to 1,

$$
E_n(\theta) - E_n(\tilde{\theta}) > 0. \tag{2}
$$

This completes the proof of Theorem **??**.
